# Obstructive sleep apnea, myocardial perfusion and myocardial blood flow: A study of older male twins

**DOI:** 10.1371/journal.pone.0278420

**Published:** 2022-11-30

**Authors:** Viola Vaccarino, Amit J. Shah, Valeria Moncayo, Jonathon A. Nye, Marina Piccinelli, Yi-An Ko, Xin Ma, Nancy Murrah, Lucy Shallenberger, Emily Driggers, Nour Jajeh, Ammer Haffar, Omar Al-Abboud, Paolo Raggi, Martica H. Hall, Richard P. Sloan, Jack Goldberg, Nicholas L. Smith, Ernest V. Garcia, Arshed A. Quyyumi, J. Douglas Bremner, Donald L. Bliwise

**Affiliations:** 1 Department of Epidemiology, Rollins School of Public Health, Emory University, Atlanta, Georgia, United States of America; 2 Department of Medicine, Division of Cardiology, Emory University School of Medicine, Atlanta, Georgia, United States of America; 3 Atlanta Veterans Affairs Health Care System, Decatur, Georgia, United States of America; 4 Department of Radiology and Imaging Sciences, Emory University School of Medicine, Atlanta, Georgia, United States of America; 5 Department of Biostatistics and Bioinformatics, Rollins School of Public Health, Emory University, Atlanta, Georgia, United States of America; 6 Mazankowski Alberta Heart Institute, University of Alberta, Edmonton, Alberta, Canada; 7 Department of Psychiatry, School of Medicine, University of Pittsburgh, Pittsburgh, Pennsylvania, United States of America; 8 Department of Psychiatry, College of Physicians and Surgeons, Columbia University, New York, New York, United States of America; 9 Seattle Epidemiologic Research and Information Center, Office of Research and Development, United States Department of Veterans Affairs, Seattle, Washington, United States of America; 10 Department of Epidemiology, University of Washington, Seattle, Washington, United States of America; 11 Department of Psychiatry and Behavioral Sciences, Emory University School of Medicine, Atlanta, Georgia, United States of America; 12 Department of Neurology, Emory University School of Medicine, Atlanta, Georgia, United States of America; University of Bologna, ITALY

## Abstract

**Background:**

Obstructive sleep apnea (OSA) has been associated with incidence of cardiovascular disease and with nocturnal angina, but evidence of a link with coronary atherosclerosis and myocardial ischemia is limited and previous studies may have been affected by selection bias or unmeasured confounding factors.

**Methods:**

We performed overnight polysomnography in 178 older male twins. The Apnea/Hypopnea Index (AHI) was calculated to assess OSA from the overnight sleep evaluation. AHI ≥15 was used as indicator of moderate/severe OSA. The following day, twins underwent myocardial perfusion imaging with [^82^Rb]-chloride positron emission tomography. Quantitative and semiquantitative measures of myocardial perfusion and absolute myocardial blood flow were obtained.

**Results:**

The mean age was 68 years and 40% of the sample had an AHI≥15, which indicates moderate to severe OSA. Abnormal myocardial perfusion, both with stress and at rest, was more common in twins with elevated AHI. After adjusting for clinical, lifestyle and behavioral factors, and previous history of cardiovascular disease, twins with AHI ≥15 had 3.6 higher odds (95% CI, 1.5–8.9) of an abnormal total severity score, defined as a score ≥100, and for each 5-point increment in AHI, the odds of abnormality increased by 20% (95% CI, 7%-34%). Twin pairs where both twins had OSA exhibited the greatest risk. There were no differences in measures of ischemia and absolute myocardial blood flow and flow reserve by AHI status.

**Conclusions:**

OSA is associated with myocardial perfusion abnormalities that suggest prior subclinical myocardial scarring or infarction. Early environmental factors that affect both twins equally may play a role and should be further explored.

## Introduction

Sleep disturbance has emerged as a consistent and independent risk factor for coronary heart disease (CHD) across a range of populations [1, 2]. Obstructive sleep apnea (OSA), in particular, has been associated with a 2–3 fold higher risk of incident CHD and stroke [3–5], and its prevalence in the population continues to grow [5, 6]. Recurrent interruptions of ventilation during sleep with a fall in blood oxygen saturation, together with sleep fragmentation and activation of the sympathetic nervous system, may cause oxidative stress, endothelial dysfunction and systemic inflammation, all of which have been linked to progression of atherosclerosis [5, 7]. Not surprisingly, a number of studies have reported that patients with OSA, compared with controls without OSA, have a higher prevalence of markers of atherosclerosis in peripheral vascular beds, such as higher carotid intima-media thickness and peripheral arterial stiffness [8–11]. Many studies have also linked OSA to measures of left ventricular dysfunction and remodeling [12]. However, studies of the relationship of OSA with atherosclerosis in the coronary circulation are more limited [13–15]. Similarly, even though OSA can trigger nocturnal angina and ST-segment depression [16–18], there are scarce and inconsistent data linking it to objective measures of myocardial injury or ischemia [19–21]. These inconsistencies may derive from limitations of previous research, including small samples and inclusion of patients referred for sleep and/or cardiac evaluation, potentially introducing referral bias. Furthermore, virtually all the common risk factors for CHD are also risk factors for OSA [1, 5, 7], leaving open the possibility of unaccounted confounding. The fact that no randomized trial of treatment of OSA has shown a benefit with respect to hard cardiovascular endpoints casts further doubt on a causal relationship between OSA and CHD [5].

To address these gaps in knowledge, we conducted a study in a well-phenotyped sample of twins to examine the association of objective measures of OSA, obtained through overnight polysomnography, with objective measures of myocardial perfusion and blood flow, using myocardial positron emission tomography (PET). OSA is two times more common in men (34%) than in women (17%) [5, 6], and some [3, 4], but not all [4, 21], studies have found that the association of OSA with CHD incidence is most pronounced in older men, making this a group relevant to study. Our hypothesis was that, among older men, polysomnographic indicators of OSA are associated with objective indices of perfusion deficit and ischemic heart disease detected by myocardial PET imaging.

## Methods

### Study sample

**Fig 1:**
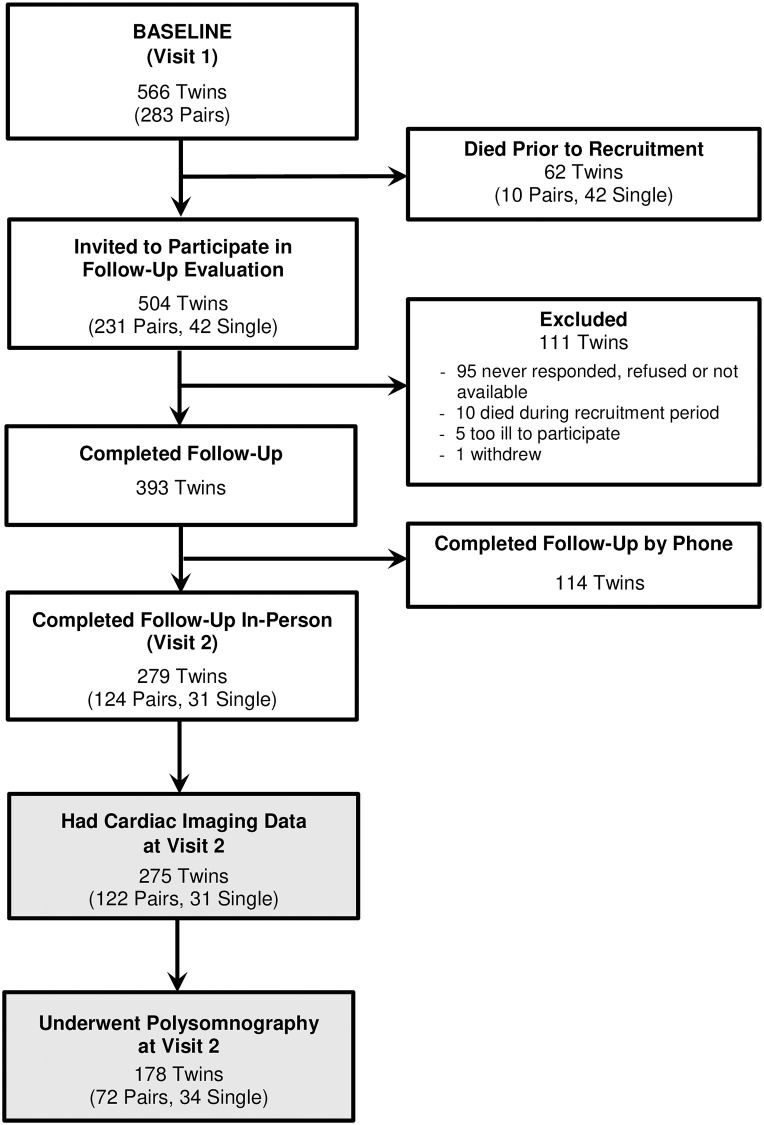
Participant flow diagram and construction of the analytical sample.

The Emory Twin Study [22, 23] included twins selected from the Vietnam Era Twin (VET) Registry, a large national sample of adult male twins who served on active duty during the Vietnam war era (1964–1975) [24]. Participants in the initial visit, completed between 2002 and 2010, included 283 monozygotic (MZ) and dizygotic (DZ) twin pairs where at least one member of the pair had PTSD or major depression along with control pairs without these conditions; twin pairs were excluded if either member of the pair had a history of cardiovascular disease at the baseline survey [25]. Of these, 275 twins completed the in-person second evaluation and had complete PET cardiac imaging data, as previously detailed [22]. Beginning in March 2017, twins also underwent one night of polysomnography for sleep evaluation. In total, 178 twins (48 MZ pairs and 24 DZ pairs, and 34 unpaired twins) had both polysomnography data and PET perfusion imaging data, and represent the sample for the current study (Fig 1).

Twin pairs participated together on the same day to minimize measurement error. They were admitted overnight in the Emory Sleep lab facility on the day prior to the PET scan, and were instructed to abstain from smoking, alcohol or caffeinated beverages. All twins signed a written informed consent, and the Emory University institutional review board approved the study.

### In-lab polysomnography

Twins underwent overnight full polysomnography (PSG) using an Embla/Natus (Pleasanton, CA) N7000 digital PSG system with Remlogic software in the Emory Sleep Center to derive measures of sleep-disordered breathing in a controlled environment. They used private rooms and were allowed to elect bedtimes and wakeup times of their own choosing but consistent with their home schedule. Procedures followed the guidelines of the American Academy of Sleep Medicine (AASM), version 2.2 [26]. Respiration was recorded with both nasal airflow pressure transducers and thermocouples placed adjacent to the mouth (for oral breathing). Respiratory effort was recorded with dual channel respiratory inductive plethysmography, and finger pulse oximetry was worn throughout the recording. After the night was completed, PSGs were scored in 30 second epochs by a single Registered Polysomnographic Technologist who was blind to all clinical data. OSA episodes were scored following current AASM guidelines for apnea (≥ 90% drop in oronasal sensor signal excursion for ≥10 secs) and hypopnea (≥ 30% drop in oronasal sensor for ≥ 10 secs accompanied by ≥ 4% drop in oxygen saturation) [26]. Apneas and hypopneas were summed and divided by the total sleep time to yield an Apnea/Hypopnea Index (AHI), which quantifies the number of apnea and hypopnea events per hour of sleep across the entire night. The AHI represented our primary measure of OSA; an AHI ≥15 is considered an indication of at least moderate OSA [27].

We also calculated other secondary indicators of OSA from the PSG. Computation of a Respiratory Disturbance Index (RDI) included hypopneas meeting oronasal criteria not necessarily accompanied by oxygen drops of ≥ 4% but with electroencephalographically defined arousals. We analyzed two measures of hypoxic burden derived from the Remlogic software: a) an oxygen desaturation index (ODI), defined as the number of desaturations ≥ 4% per hour of sleep, and b) the cumulative proportion (%) of sleep time spent with oxygen saturation < 90%.

Two participants were not able to undergo PSG at Emory due to their inability to abstain from continuous positive airway pressure (CPAP) use during sleep. For these two individuals we used the results of a previous PSG they had performed within two years of the Emory visit.

### Measurement of myocardial perfusion and absolute coronary flow

As previously described [22], twins underwent myocardial perfusion imaging with PET using [^82^Rb]-chloride at rest and following pharmacologic stress using regadenoson, an analogue of the A2A adenosine receptor, during a single imaging session using a Biograph PET/CT (Siemens, Knoxville, Tennessee) in 3-dimensional mode. All medications were held the morning of the PET scan. Blood pressure and heart rate were recorded before the pharmacological stress test, and every minute for 4 minutes during the test and the recovery period. The peak rate-pressure product during pharmacological stress was calculated as the maximum systolic blood pressure times the maximum heart rate.

Myocardial perfusion was quantified using the Emory Cardiac Toolbox (Syntermed Inc., Atlanta, Georgia), a computerized tool that provides automated (operator-independent) quantitative assessment of perfusion with established validity and reproducibility [28]. Using this method, the three-dimensional tracer uptake distribution in the left ventricular myocardium was synthetized onto a two-dimensional polar map. A total severity score was computed as an automated, quantitative indicator of total perfusion deficit, according to published methodology [28, 29]. The total severity score takes into account both size and severity of the abnormality, and is calculated by summing the number of standard deviations of pixels in the stress map exceeding 2.5 standard deviations below the mean normal levels for all pixels in the defect (based on the mean values of a normal file). We calculated the percentage of participants with a score of 100 or greater, which corresponded to the upper quartile of the distribution in our sample, and was previously found to have prognostic significance [30]. The total severity score was our main variable of interest. The computerized algorithm also produced a severity score at rest and an index of reversibility (reversibility severity score), as well as a measure of ischemic burden (% of left ventricle) quantified using the 17-segment model [29]. Images were also interpreted visually by a nuclear medicine physician (VM) blinded to other study data, with calculation of semiquantitative summed stress, rest and difference scores in a conventional fashion [31]. The percentage of abnormal myocardium was computed by dividing the summed stress score by 68 (maximum score of 4 for each of 17 myocardial segments in the polar map) and multiplying it by 100, and ischemia was defined as a summed difference score ≥4 [31]. Left ventricular ejection fraction and left ventricular mass were calculated from gated myocardial perfusion images [28].

We performed absolute myocardial blood flow quantitation for the assessment of myocardial flow reserve, an index of coronary vasodilator capacity that is an accepted measure of coronary microvascular function if there are no obstructive coronary lesions [31]. Measurements of absolute myocardial blood flow at rest and during peak hyperemia were obtained for each myocardial segment using clinically accepted models of absolute myocardial blood flow corrected for radiotracer extraction fraction [32]. An overall measure of myocardial flow reserve for the entire myocardium was calculated as the ratio of maximum absolute flow during hyperemia to absolute flow at rest. We also considered the three major coronary territories separately: left anterior descending, left circumflex, and right coronary artery.

### Other measurements

Twins received a thorough assessment including medical history, including history of OSA, sociodemographic information, health behaviors, blood pressure and anthropometric data, as previously described [22, 23]. Physical activity was measured using the Baecke Questionnaire of Habitual Physical Activity [33]. History of cardiovascular disease that might have occurred from the initial visit was defined as previous myocardial infarction, stroke or coronary revascularization procedures. The Structured Clinical Interview for Diagnostic and Statistical Manual of Mental Disorders, 4^th^ Edition (DSM-IV) [34] was administered to assess lifetime history of major depression, posttraumatic stress disorder and substance abuse. Service in Southeast Asia was determined from military records via the VET Registry. Zygosity information was assessed by DNA typing as previously described [35].

### Statistical analysis

Analyses were first conducted considering twins as individuals, using generalized estimating equation (GEE) models with a random intercept for each twin pair [36]. Our main indicator of OSA was AHI categorized at the cut point of 15 (an AHI ≥15 indicates at least moderate OSA [27]). We first summarized participants’ characteristics based on OSA status and then compared PET data of myocardial perfusion and absolute myocardial blood flow quantitation across the two categories of AHI.

The main outcome measure in the analysis was a two-level total severity score of <100 or ≥100, because of the non-normal distribution of this variable. Using GEE models, the association between OSA and the outcome was first examined by comparing the proportion of twins with a total severity score ≥100 across tertiles of AHI and other indices of OSA and hypoxic burden. Next, we performed multivariable analysis for the relation of both binary AHI (at the cut point of 15) and continuous AHI (per 5-point increments), with a total severity score ≥100 as the dependent variable. Analyses were adjusted sequentially for a set of factors selected a priori, including age, history of cardiovascular disease and cardiovascular risk factors, including diabetes, hyperlipidemia, measured systolic blood pressure and measured waist circumference, and lifestyle factors, including history of smoking (current, past or never), alcohol use (number of alcoholic drinks in average week in past 30 days) and physical activity (Baecke score). In a subsequent model, we adjusted for lifetime history of major depression, lifetime history of PTSD, and current use of antidepressant medications.

To verify the robustness of our results, we also considered the total severity score as a continuous variable. Because this variable had a cluster (47%) at 0 and a skewed distribution for the non-zero portion, it was considered as a two-part mixture distribution and analyzed using a two-part nonlinear mixed-effects model with random intercept for twin pair. This model fits the difference in total severity score as a continuous variable for the nonzero values of the score, while at the same time fitting the probability of a score >0 (PROC NLMIXED in SAS) [37].

Next, we conducted an analysis within twin pairs discordant for AHI and between (across) pairs. There were only 27 twin pairs discordant for AHI ≥15 vs. <15; thus, the within-pair analysis treated AHI as a continuous variable which included 70 discordant pairs (at least 5 point-difference between brothers). The within-twin pair parameter represented the departure of each twin from the twin pair average, while the between-twin-pair parameter represented the twin-pair average for the independent variable [36]. The within-pair associations are inherently controlled for demographic, shared familial and early environmental influences; environmental factors during the examination day are also controlled by design since twin pairs were examined together. If the within-pair estimates are smaller than those seen when twins are analyzed as separate individuals, and if the between-pair estimate is significant (comparing different families) this suggests familial effects on the association [38]. To assess potential shared genetic influence between AHI status and myocardial perfusion, within-pair associations were examined in MZ and DZ pairs separately and the interaction by zygosity was tested. However, because of the small sample of pairs by zygosity, this analysis was exploratory. Because GEE modeling can cause incorrect estimation of within-pair effects [36], we used generalized linear mixed models with logit link and adaptive quadrature estimation which is based on the maximum likelihood method (PROC GLIMMIX in SAS).

Missing data were rare for all variables in the analysis (<5%), thus all available data were used without imputation. A two-sided p-value of less than 0.05 was considered as statistically significant, and 95% confidence intervals (CI) were calculated from model parameters. All statistical analyses were performed using SAS, version 9.4 (SAS Institute, Cary, NC).

## Results

### Sample characteristics and bivariate associations

The prevalence of OSA was high; of the 178 twins in the study, 72 (40.4%) had an AHI of 15 or above, denoting moderate/severe OSA. The mean age was 68 years and did not differ by OSA category (AHI <15 or AHI ≥15) (Table 1). Of those with AHI ≥15, only 37.5% had a previous history of OSA, and only 22.5% used CPAP at home. sociodemographic factors and lifestyle behaviors, as well as psychiatric history and current medications, were similar. As expected, however, twins with elevated AHI tended to be heavier and to have a higher blood pressure. A past clinical history of cardiovascular disease was infrequent (especially a past history of myocardial infarction), but tended to be more common among twins with OSA (AHI ≥15). A history of revascularization procedures and a history of angina pectoris were also more frequent among participants with OSA.

**Table 1:** Sociodemographic factors, military service, lifestyle, and cardiovascular disease risk factors in twins with and without obstructive sleep apnea.

	No OSA (AHI <15) N = 106	OSA (AHI ≥15) N = 72
**Sociodemographic Factors**		
Age, yrs, mean (SD)	68.4 (2.4)	68.6 (2.3)
Non-white, %	5.7	1.4
Married, %	72.6	75.0
Years of education, mean (SD)	14.0 (2.3)	13.8 (2.4)
Employed full time, %	22.6	19.4
Service in Southeast Asia, %	38.7	43.0
**Lifestyle Factors**		
Cigarette Smoking, %		
Never	34.9	36.1
Former	50.9	52.8
Current	14.2	11.1
Physical Activity (Baecke Score)	8.2 (1.2)	7.6 (1.3)
Number of alcoholic drinks in average week, past 30 days, mean (SD)	5.8 (15.2)	5.0 (8.6)
**History of Obstructive Sleep Apnea**		
Ever told he had obstructive sleep apnea, %	10.4	37.5
Ever treated with CPAP, %	7.5	30.6
Currently using CPAP at home, %	3.8	22.5
**Cardiovascular Risk Factors and Cardiovascular History**		
History of cardiovascular disease, % ^a^	8.5	18.0
History of myocardial infarction, %	0.9	8.4
History of stroke, %	2.8	4.2
History of revascularization procedures, %	10.4	24.3
History of angina pectoris, %	10.4	24.3
History diabetes, %	19.8	22.2
History of hyperlipidemia, %	60.9	69.4
History of hypertension, %	47.2	75.0
BMI, mean (SD)	28.2 (3.4)	31.5 (4.4)
Waist circumference, cm (SD)	103 (11)	111 (11)
Mean systolic blood pressure, mm Hg, mean (SD)	139 (20)	143 (18)
Mean diastolic blood pressure, mm Hg, mean (SD)	78.3 (11.1)	82.0 (12.2)
**Psychiatric Diagnoses (Lifetime)**		
Major Depression, %	22.6	19.4
PTSD, %	24.5	23.6
Alcohol Abuse (with or without dependence), %	28.3	14.4
Drug Abuse (with or without dependence), %	14.1	5.6
**Current Medications**		
Aspirin, %	43.4	51.4
Statins, %	48.1	62.5
Beta-blockers, %	17.9	36.1
ACE Inhibitors, %	18.9	31.9
Antidepressants, %	17.0	15.3

All data are percentages unless otherwise indicated.

Abbreviations: OSA: obstructive sleep apnea; SD: standard deviation; CPAP: continuous positive airway pressure; BMI: body mass index; PTSD: Posttraumatic Stress Disorder; ACE: angiotensin converting enzyme.

^a^ History of cardiovascular disease included previous myocardial infarction, stroke or revascularization procedures.

There were no significant differences by OSA status in hemodynamic factors, before or during the hyperemia test, although the resting heart rate was higher in twins with OSA compared with those without OSA (Table 2). Quantitative indicators of fixed perfusion deficit were more frequent in twins with OSA compared with those without OSA. The mean total severity score was twice as high in twins with OSA (120.4) vs. those without OSA (58.4, p = 0.01), and 37.5% of twins with OSA had a total severity score ≥100, compared with 17.9% of twins without OSA (p = 0.004). The difference in total severity score was driven by the rest severity score, which was higher in twins with OSA than those without (4.4 vs. 34.3, p = 0.02), while there were no differences in quantitative indices of ischemia (reversibility severity score and ischemic burden). The results of the semiquantitative visual analysis confirmed these findings (Table 2). There were no significant differences in absolute myocardial blood flow and myocardial flow reserve by OSA status, although there was a tendency towards a lower myocardial flow reserve in twins with OSA, overall and in each of the three major coronary territories (Table 2).

**Table 2:** Myocardial perfusion imaging data in twins with and without obstructive sleep apnea.

	No OSA (AHI <15) N = 106	OSA (AHI ≥15) N = 72	P
	Mean [95% CI] or n (%)		
**Stress Test Hemodynamic**			
Resting systolic blood pressure, mm Hg	144 [141–148]	148 [143–152]	0.22
Resting diastolic blood pressure, mm Hg	78.0 [75.7–80.3]	78.5 [75.7–81.3]	0.81
Resting heart rate, beat/min	63.2 [60.8–65.5]	68.0 [64.9–71.2]	0.01
Maximum systolic blood pressure, mm Hg	132 [128–136]	135 [131–140]	0.31
Maximum diastolic blood pressure, mm Hg	67.2 [65.4–69.1]	68.8 [66.5–71.0]	0.30
Maximum heart rate, beat/min	88.3 [86.0–90.6]	87.4 [84.5–90.4]	0.62
Maximum rate-pressure product, beat x mm Hg/min per 1000	11.7 [11.1–12.3]	11.8 [11.2–12.4]	0.75
**Myocardial Perfusion**			
Quantitative automated analysis			
Total severity score	58.4 [37.2–79.5]	120.4 [74.8–165.9]	0.01
Total severity score ≥ 100, n (%)	19 (17.9)	27 (37.5)	0.004
Rest severity score	4.4 [0.9–7.9]	34.3 [8.7–60.0]	0.02
Ischemic burden, % LV	1.2 [0.7–1.7]	1.4 [0.8–2.0]	0.55
Reversibility severity score	15.4 [5.2–25.6]	12.3 [1.1–23.5]	0.68
Semiquantitative visual analysis			
Summed Stress Score	1.6 [0.9–2.3]	2.9 [1.7–4.2]	0.06
Summed Rest Score	0.3 [0.1–0.5]	2.0 [0.9–3.1]	0.004
Total myocardium abnormal, % LV	2.3 [1.3–3.3]	4.3 [2.5–6.2]	0.06
Ischemia (summed difference score ≥4) (%)	15 (14.2)	8 (11.1)	0.58
Rest left ventricular ejection fraction, %	65.3 [63.7–66.9]	63.9 [61.6–66.3]	0.31
Rest left ventricular mass, g	104 [101–108]	108 [103–113]	0.15
**Absolute Myocardial Blood Flow Quantitation**			
Stress myocardial blood flow, mL/min/g, whole myocardium	1.49 [1.40–1.58]	1.48 [1.36–1.61]	0.90
Rest myocardial blood flow, mL/min/g, whole myocardium	0.72 [0.68–0.76]	0.79 [0.68–0.90]	0.19
Myocardial Flow Reserve, mL/min/g, whole myocardium	2.09 [2.00–2.17]	2.03 [1.90–2.15]	0.39
Myocardial Flow Reserve, mL/min/g, LAD	2.14 [2.05–2.23]	2.06 [1.93–2.19]	0.29
Myocardial Flow Reserve, mL/min/g, LCX	2.08 [1.99–2.16]	2.01 [1.89–2.14]	0.40
Myocardial Flow Reserve, mL/min/g, RCA	2.03 [1.94–2.12]	1.99 [1.87–2.12]	0.66

Abbreviations: OSA: obstructive sleep apnea; AHI: Apnea Hypopnea Index; CI: confidence interval; LV: left ventricle; LAD: left anterior descending coronary artery; LCX: left circumflex coronary artery; RCA: right coronary artery.

**Fig 2:**
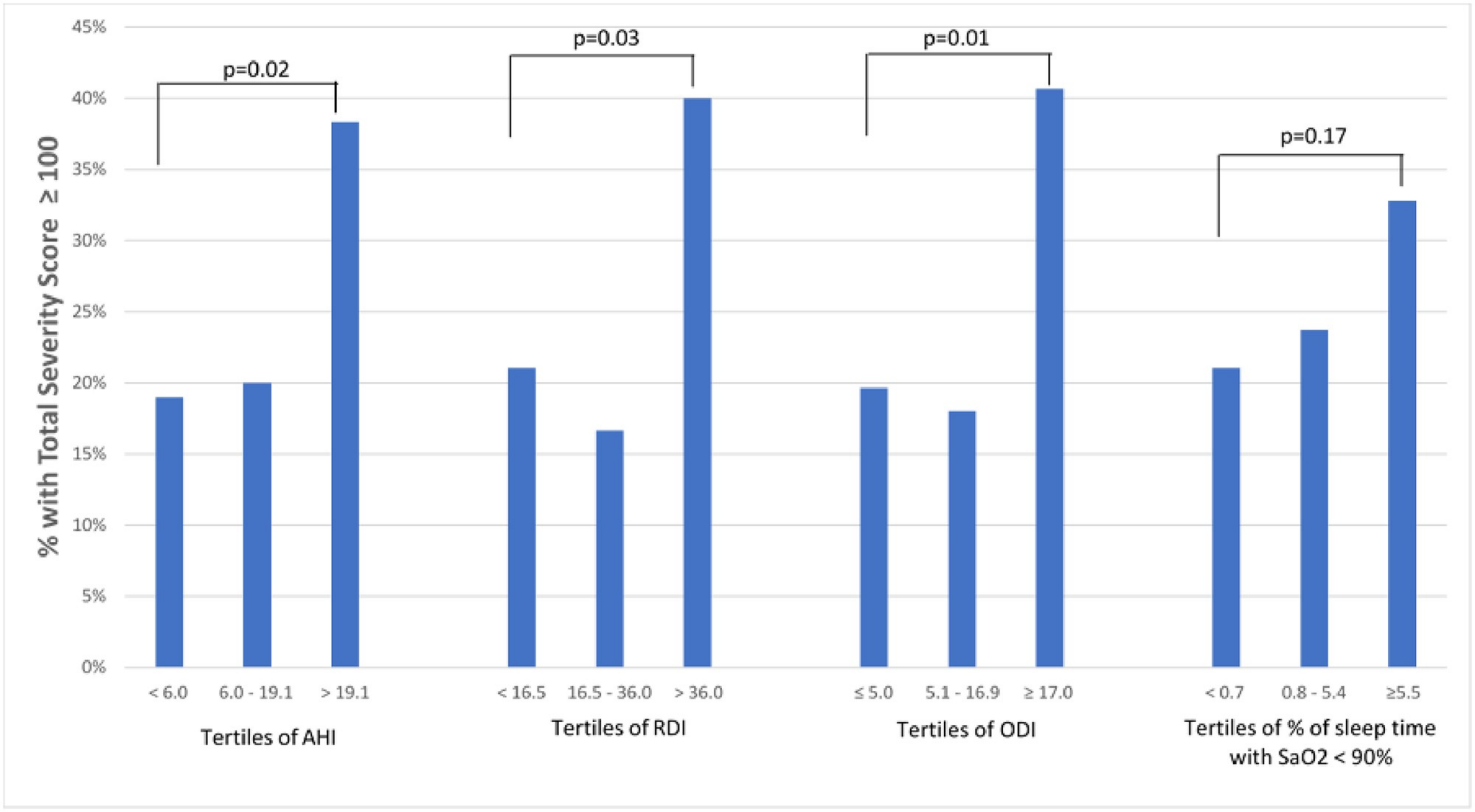
Association of a total severity score ≥100, a measure of abnormal myocardial perfusion obtained using positron emission tomography myocardial perfusion imaging, with indicators of obstructive sleep apnea classified in tertiles of their distribution, including the Apnea/Hypopnea Index (AHI), the Respiratory Disturbance Index (RDI), the oxygen desaturation index (ODI), and the cumulative proportion (%) of sleep spent with oxygen saturation (SaO2) < 90%. P values test the difference between first and third tertile.

The relation of OSA with perfusion deficit persisted with OSA indicators other than AHI (Fig 2). Across tertiles of their distribution, RDI, ODI, and the percent of sleep time with oxygen saturation <90%, in addition to AHI, were all related to total severity score, with the highest tertile showing a significantly higher prevalence of a total severity score ≥100.

### Multivariable analysis

Before adjusting for covariates, twins with OSA (AHI ≥15) had almost 3-fold higher odds of a total severity score ≥100 than those without OSA. This association was not diminished after adjusting for clinical, lifestyle and psychological factors (Table 3). The association remained also when considering AHI as a continuous variable. In the fully adjusted model, for each 5-unit increment in AHI, there was 20% higher odds of having a total severity score ≥100.

**Table 3:** Multivariable analysis of the relationship between the Apnea-Hypopnea Index (AHI), both as a dichotomous variable (AHI ≥15) and as a continuous variable, and total severity score ≥100, in the overall sample with twins treated as individuals.

	Dichotomous AHI (AHI ≥ 15 vs AHI <15)				Continuous AHI (per 5-unit increase)		
Outcome: Total Severity Score ≥100	n	OR	[95% CI]	P	OR ^d^	[95% CI]	P
Unadjusted	178	2.75	[1.38–5.48]	0.004	1.13	[1.04–1.23]	0.005
Adjusted for age and clinical risk factors ^a^	176	3.18	[1.42–7.12]	0.005	1.18	[1.07–1.31]	0.001
Adjusted for all above and lifestyle factors ^b^	169	3.26	[1.31–8.09]	0.01	1.20	[1.07–1.34]	0.001
Adjusted for all above and psychological factors ^c^	169	3.61	[1.47–8.88]	0.005	1.20	[1.07–1.34]	0.001

^a^ History of cardiovascular disease, diabetes and hyperlipidemia, measured systolic blood pressure and waist circumference.

^b^ History of smoking (current, past or never), alcohol use (number of alcoholic drinks in average week in past 30 days) and physical activity (Baecke score).

^c^ Lifetime history of major depression, lifetime history of PTSD, and current use of antidepressant medications.

^d^ AHI is expressed in percentage point units of time spent in apnea/hypopnea per sleep hour, averaged across the whole night.

When considering the total severity score as a continuous variable for values > 0, in a two-part nonlinear mixed-effects model, results remained consistent. In this model, for nonzero values for the severity score, twins with AHI ≥ 15 had a 65.4% higher average total severity score than those with AHI <15 (95% CI, 6.7%-156%, p = 0.02). The model also fitted the probability of a score > 0; twins with AHI ≥15 had 2.1 higher odds of a total severity score > 0 compared to twins with AHI <15 (95% CI, 1.1–3.9, p = 0.02).

### Within-pair and between-pair analysis

When comparing twins within the 70 pairs where brothers were discordant for AHI (at least 5-point difference), the association was only slightly attenuated, although it was no longer significant, with similar estimates by zygosity (Table 4). Comparing twin pairs (or families), a 5-point higher pairwise mean AHI was associated with a 19% higher pairwise mean odds of a total severity score ≥100. Furthermore, when considering AHI as a dichotomous variable, there was a dose-response relationship, such that, compared to twin pairs where neither twin had OSA (AHI ≥15), pairs where 1 twin had OSA (OR = 1.7), and especially pairs where both twins had OSA (OR = 5.4), had an elevated mean OR for a total severity score ≥100 (Table 4).

**Table 4:** Within-pair and between-pair relationships between the Apnea-Hypopnea Index (AHI) as a continuous variable and total severity score ≥100, in twins discordant for AHI (at least 5-point difference). The pairwise analysis is also shown for AHI status as a categorical variable.

**Within-Pair Analysis**					
	**n**	**OR Between Discordant Twins**	**[95% CI]**	**P**	
All discordant pairs for continuous AHI (per 5-unit increase)	70 pairs	1.10	[0.88–1.36]	0.39	
MZ	47 pairs	1.12	[0.84–1.50]	0.43	
DZ	23 pairs	1.08	[0.76–1.52]	0.35	
**Pairwise Analysis**					
	**n**	**Mean OR for Twins in Pair**	**[95% CI]**	**P**	**P**
All twins, continuous AHI (per 5-unit increase)	72 pairs, 34 single	1.19	[1.03–1.38]	0.02	
Neither twin with AHI ≥15	29 pairs	Ref.	--	--	0.02
1 twin with AHI ≥15	27 pairs	1.67	[0.49–5.67]	0.40	
Both twins with AHI ≥15	16 pairs	5.36	[1.36–21.16]	0.01	

## Discussion

In this study of older Vietnam Era Veteran twins, we found a robust association between polysomnographic indicators of OSA, primarily defined as an AHI ≥15, and objective indices of myocardial perfusion deficit using PET imaging, even after adjusting for cardiovascular risk factors and other comorbidities. The total severity score, a quantitative measure of myocardial perfusion deficit, was substantially increased among twins with OSA, but there were no significant differences in ischemia or changes in absolute myocardial blood flow during the hyperemia test. This suggests that the association of OSA with myocardial perfusion deficit was driven by fixed defects that indicate prior myocardial infarction or scar. The most significant findings were at the pair level, such that a higher AHI of the pair was associated with a higher perfusion deficit. When both twins in a pair had an elevated AHI, their likelihood of having myocardial perfusion deficit increased more than fivefold. This suggests that non-genetic familial factors influence both OSA and CHD; these factors warrant further investigation.

OSA is a common disorder and an important and growing public health problem [1]. OSA has been clearly associated with many different forms of cardiovascular disease events, including hypertension, stroke, CHD, heart failure and atrial fibrillation [5], as well as with a wide variety of abnormalities in cardiac structure and function [12]. Evidence of a link between OSA and objective measures of atherosclerosis in the coronary bed has been more limited but overall has suggested an association of OSA with coronary plaque burden and plaque composition [12]. For example, in a sample of 19 patients with stable CHD evaluated with 3-dimensional intravascular ultrasound, Turmel et al. found a higher atherosclerotic plaque volume in those with more frequent episodes of obstructive sleep apnea/hypopnea [13]. In another small study, the frequency of noncalcified or mixed plaques was higher in patients with OSA than in non-OSA controls undergoing multidetector-row helical computed tomography [14]. A larger study in China reported an association between OSA and measures of plaque volume and composition using computerized tomography coronary angiography [15]. Our results of an association of OSA with myocardial perfusion abnormalities are consistent with these previous investigations linking OSA to measures of atherosclerotic burden in the coronary circulation, as well as with observations of an association of OSA with atherosclerosis in non-coronary territories [8–11].

In our study, the association of OSA with myocardial perfusion deficit was driven by resting abnormalities. There were no associations of OSA with indicators of reversibility and myocardial flow reserve. This could be explained by the fact that ischemic abnormalities were examined the following day, rather than during the night in conjunction with acute OSA episodes. Nonetheless, these results are in line with several studies that have questioned whether OSA can trigger myocardial injury or ischemia, despite its reported associations with nocturnal chest pain and ST-segment changes [16–18]. It was suggested that patients with established coronary atherosclerosis, but not those without this condition, may be prone to ischemic changes during OSA episodes [18]. However, a study of patients with known CHD and moderate-to-severe OSA undergoing polysomnography, failed to show evidence of myocardial injury during sleep, assessed by a third-generation troponin T assay [19]. Even in community studies the relationship between OSA and myocardial injury has also been variable. In a sample of individuals from the general population, the presence of detectable high-sensitivity cardiac troponin T increases in proportion to OSA severity, but the association disappeared after accounting for the higher prevalence of cardiovascular risk factors among those with OSA [20]. In another community study, OSA was related to high-sensitivity cardiac troponin T in women but not in men [21]. In a sample of 36 study participants with AHI >15 but otherwise healthy, evaluated with myocardial contrast echocardiography with dipyridamole stress, Butt et al. demonstrated a reduction in myocardial flow reserve compared with controls [39], however, they also showed that much of the flow abnormalities found in OSA patients were already present at rest, as in our study. Taken together with existing evidence, our results do not lend support to the possibility that OSA can induce myocardial ischemia and potentially trigger acute coronary syndromes, at least among individuals from the community with limited or no evidence of previous CHD. Although it may sound contradictory that OSA can be involved in atherosclerosis progression but not in myocardial ischemia, a possible explanation is that chronic intermittent hypoxia, a feature of OSA, may favor protective mechanisms such as ischemic preconditioning in the myocardium and development of coronary collaterals, reducing ischemic burden [40, 41]. More research is warranted, however, to explore this further.

CHD and OSA share most common risk factors [1, 5, 7], and it is difficult to account for all these shared influences. Our study suggests that the association between OSA and coronary atherosclerosis is driven, at least in part, by familial factors. This suggests that both conditions share similar early life precursors such as environmental exposures, health behaviors, socioeconomic and parental influences that may shape health beginning at a young age. These factors can be important antecedents of both CHD and other chronic conditions associated with CHD, which could extend to sleep-disordered breathing. Our findings provide one explanation for why randomized trials of OSA treatment have generally failed to demonstrate a benefit towards improving cardiovascular outcomes [5, 42–44], although OSA symptoms and quality of life [42] and left ventricular diastolic function [45] may improve. An important implication of our results is that the risk of both chronic conditions (CHD and OSA) could be mitigated by a primordial prevention approach beginning early in the lifecourse.

Our sample was mostly White and restricted to older male veterans, thus it remains unclear the extent to which our results are generalizable to other groups. Future studies should include women and samples that are ethnically diverse. This should also allow the examination of potential differences in results in various demographic groups. In addition, the cross-sectional nature of this study precludes our ability to infer causality. Our measures of ischemia and myocardial blood flow were taken the day after the overnight polysomnographic evaluation, and thus we cannot exclude the possibility of ischemic abnormalities in conjunction with acute OSA episodes. Furthermore, the sample size was small, which limited the statistical power to make inferences on within-pair effects and the influence of genetic factors. However, our study is strengthened by the co-twin study design which allows to uncover familial and early environmental influences [38]. Our study also has the important advantage of using established measures of both OSA though in lab polysomnography and of myocardial perfusion using PET imaging.

## Conclusion

Our study in a sample of twins predominantly free of a previous history of CHD, provides compelling evidence that OSA is associated with coronary artery disease burden but not with myocardial ischemia. Our study also suggests that OSA and coronary atherosclerosis partially share roots in early environmental and familial factors. These data support the notion that primordial prevention of cardiovascular risk beginning in youth should achieve the best results for promoting not only cardiovascular health, but also associated comorbidities like obstructive sleep apnea.
